# Does Trauma Exacerbate Criminal Behavior? An Exploratory Study of Child Maltreatment and Chronic Offending in a Sample of Chinese Juvenile Offenders

**DOI:** 10.3390/ijerph191811197

**Published:** 2022-09-06

**Authors:** Xuening Yao, Hongwei Zhang, Ruohui Zhao

**Affiliations:** 1 Department of Sociology, Faculty of Social Sciences, University of Macau, Macau SAR 999078, China; 2 School of Humanities, Jinan University, Zhuhai Campus, Zhuhai 519070, China

**Keywords:** child maltreatment, physical abuse, chronic offending, Chinese juvenile offenders

## Abstract

(1) Background: Maltreated children are at increased risk for juvenile delinquency. Extant research has explored the effect of child maltreatment on either the initial risk of juvenile delinquency or general juvenile recidivism. However, little is known regarding the effect of child maltreatment on chronic offending. (2) Methods: Using a sample of 695 male juvenile offenders incarcerated in a centralized juvenile reformatory of the province X located in Southwest China, this study investigates both the prevalence of child maltreatment and the effect of child maltreatment on chronic offending among the juvenile offenders. Descriptive statistical analyses and multinomial logistic regression were utilized to conduct the analyses. (3) Results: A vast majority of the juvenile offenders experienced at least one type of child maltreatment. Moreover, maltreatment was generally found to be more prevalent in chronic offenders than in one-time offenders and recidivists. Results from a series of logistic regression analyses revealed that among five specific maltreatment types, only physical abuse exerted a statistically significant and positive impact on chronic offending. (4) Conclusions: The findings highlight the importance of providing early prevention and intervention programs to juvenile offenders who were physically abused in order to reduce general chronic offending as well as chronic violent offending.

## 1. Introduction

Child maltreatment is a broad concept that covers multiple aspects of children’s experiences of maltreatment [1]. It has been viewed as a significant threat to public health and social welfare [2,3]. Child maltreatment has been found to be prevalent among the adolescent population, specifically in the juvenile offender population [4,5]. A host of Western empirical studies have found a high prevalence of child maltreatment victimization among juvenile offenders [6,7,8]. For example, a vast majority of juvenile offenders, ranging from 60% to 90%, reported a history of at least one type of child maltreatment victimization, including physical abuse, emotional abuse, sexual abuse, physical neglect, and emotional neglect [7,9,10]. In China, previous studies have mainly focused on estimating the prevalence of child maltreatment victimization among adolescents in general [11,12]. Only one relevant Chinese study has explored the prevalence of child sexual abuse victimization among male juvenile offenders (21.8%) [13].

### 1.1. Research Gap

A growing body of empirical research conducted in the West and China has generally identified child maltreatment as an important risk factor related to general juvenile delinquency [14,15,16]. A handful of Western studies have also investigated the effect of child maltreatment on juvenile recidivism or reoffending behavior [17,18]. However, studies on the impact of child maltreatment on juvenile chronic offending, a type of frequently recurring offending over time, remain rather limited.

### 1.2. Aims and Justifications for the Current Study

The current study made use of data collected from a centralized provincial-level male juvenile reformatory in China to address the research gap in the existing literature by exploring: (1) the prevalence of both child maltreatment and chronic offending among juvenile offenders; (2) the holistic effect of child maltreatment and differential effects of specific maltreatment types on juvenile chronic offending in the Chinese context. It may contribute to the existent literature with the following. First, to the authors’ best knowledge, the present study is probably the first to systematically explore the effect of child maltreatment on juvenile chronic offending. Despite the extensive efforts made to explore the effect of child maltreatment on juvenile offending [14,16] or recidivism [17,18] in Western research, relevant Western studies on the impact of child maltreatment on chronic offending have been sporadic, and their findings have been inconsistent. Such studies are lacking in China. Second, nearly all published Chinese studies have assessed the prevalence of child maltreatment victimization and juvenile delinquency among school-based adolescent samples [11,19]. By making use of the juvenile offender sample, the present study provides a rare opportunity to explore the relationship between child maltreatment and juvenile chronic offending in China, a society that is culturally unique and different from the West.

## 2. Literature Review

### 2.1. Theoretical Explanations of the Relationship between Child Maltreatment and Juvenile Chronic Offending

To date, there has been no theory explicitly explaining the relationship between child maltreatment and juvenile chronic offending. However, the relationship between child maltreatment and general juvenile delinquency can well be explained by Agnew’s general strain theory and Akers’ social learning theory. One of the strain theory’s major propositions has been that the presentation of negative stimuli, such as adverse life events and child abuse victimization, is a source of strain or negative emotions for individuals who experienced such negative stimuli, which then may lead to criminal behavior as a coping strategy to the negative emotions [20]. Agnew argued that not all strains inevitably lead to crime, and certain strains should be prioritized because they are found to be more likely to trigger crime. Strains, which are considered (a) high in magnitude, (b) unfair or unjust, (c) to be associated with low social control, or (d) to create pressure or incentive for criminal coping are identified as the strains that are the most likely to cause crime [21]. Drawing on these characteristics, Agnew developed a list of those strains conducive to crime, including parental rejection, erratic supervision, harsh discipline, child abuse and neglect, and criminal victimization [21]. According to this theory, child maltreatment is clearly a source of strain that may lead to juvenile delinquency. Chronic criminal offenses committed by maltreated children may be considered a strategy adopted by the children to cope with the negative emotions caused by their family members’ repeated abusive behavior.

Social learning theory specifies the mechanisms and processes of deviant behavior learning. The basic premise of the theory is that criminal behavior is initially learned through social interaction, such as direct imitation or modeling of other people’s behavior, and that continued criminal behavior is determined by differential reinforcement, which comprises the relative rewards and punishments following the act [22]. Reinforcement of criminal behavior can be either direct by rewards following one’s own initial criminal act(s) or indirect by simply observing another’s criminal behavior being rewarded [22]. Social learning theory provides a theoretical rationale for the relationship between child maltreatment and delinquency and later criminal involvement [22]. First, children exposed to maltreatment learn the patterns of behavior and deviant values from their abusive parents through direct imitation. This has often been called the “cycle of violence” in previous research [23]. Studies exploring this cycle initially focused mainly on the relationship between child physical abuse and the perpetuation of violence [23,24]. Subsequent studies have extended to explore the relationship between various types of child maltreatment and later criminal behaviors [25,26]. However, the detrimental effects of specific maltreatment types on criminal behavior vary; certain maltreatment types have a greater impact on general offending [27,28] or certain offending types [29,30]. Second, sustained or chronic criminal behavior committed by maltreated children may be the result of the differential reinforcement process in which delinquent behavior was either directly rewarded by monetary gain following the criminal act or indirectly rewarded by observing the abusive behavior of the parents not being punished.

Both theories help explain the relationship between child maltreatment and chronic offending. Child maltreatment is considered a source of either strain or imitation that leads to criminal behavior. Compared with strain theory, social learning theory seems to place more focus on the criminal reinforcement processes and the subsequent reoffending or chronic offending.

### 2.2. Findings of Previous Empirical Studies

The term “chronic offender” was initially proposed by Wolfgang et al., in their seminal work titled *Delinquency in a Birth Cohort*. It is defined as any juvenile offender responsible for at least five offenses between the ages of 10 and 18 [31]. This unique group only accounts for a small proportion of juvenile offenders but commits a large proportion of the total criminal activities [32]. A host of prior Western research has identified a few risk factors for chronic offending, including child maltreatment, individual impulsivity, substance use, peer deviance, gang involvement, and dropping out of school [33,34].

A thorough search of the literature on the relationship between child maltreatment and chronic offending resulted in the identification of three empirical studies tapping into this relationship among either juvenile or adult offenders in Canada and the United States [35,36,37]. None of the three studies, however, simultaneously investigated the holistic effect of child maltreatment and the differential effects of specific maltreatment types on chronic offending among juvenile offenders. Moreover, only one of the studies regarded physical–emotional abuse, sexual abuse, and neglect as core predictor variables of chronic offending [35]. The remaining two studies merely measured physical abuse and sexual abuse as either control variables [36] or maltreatment subtypes as part of adverse childhood experiences [37]. Furthermore, the three studies yielded inconsistent findings regarding the effect of specific maltreatment types on chronic offending. One study found the significant effects of physical–emotional abuse and sexual abuse on chronic offending [35], and another study found the significant effects of physical abuse, emotional abuse, and physical neglect [37]. However, the third study found that neither physical abuse nor sexual abuse exerted a significant effect on chronic offending [36].

In contrast, the Chinese literature has been silent on the issue of child maltreatment as a risk factor for chronic offending among juvenile offenders. One of the reasons might be due to the low delinquency rate among general adolescent population and a lack of accessibility to juvenile offenders. A replication of the study by Wolfgang et al. [31] among 5241 adolescents in Wuhan City, China, found that the delinquency rate of Chinese adolescents was far lower than that of the United States—2% in China vs. 35% in the United States [38]. The small number of delinquents (*n* = 81) among the general adolescent sample (*n* = 5241) poses a challenge to studying not only delinquency but also chronic offending using general adolescent sample in China. A juvenile offender sample, therefore, makes it possible to conduct research on chronic offending in the Chinese context. To date, only one Chinese study employed an adult offender sample to explore the applicability of the concept of the chronic offender [39]. The study discovered findings similar to research conducted in the United States—that is, a small group of chronic offenders (7.5%) tended to commit a relatively large number of crimes (26.51%) [39]. Risk factors including child maltreatment victimization for chronic offending have not been researched in China.

## 3. Methodology

The present study is quantitative and exploratory in nature. It made use of descriptive and correlational design to explore: (1) the prevalence of both child maltreatment and chronic offending among juvenile offenders; (2) the relationship between child maltreatment and juvenile chronic offending in the Chinese context.

### 3.1. Sample

This study took place in an ethnic minority autonomous region (administratively equal to a province) located in Southwest China. According to the most recent national census, the population of this autonomous region exceeded 50 million in 2021 [40]. As one of the five ethnic minority autonomous regions in China, approximately 37.5% of the population are ethnic minorities [40]. Data collected from this region provide a rare opportunity to explore if race or ethnicity exerts an effect on juvenile chronic offending in China.

According to the 2020 Criminal Law of the People’s Republic of China, juvenile offenders between the ages of 12 and 18 years who committed designated serious crimes are held criminally liable for their crimes (Chapter 2, Article 17). The most severe punishment for them is incarceration in a juvenile reformatory. This study only worked with male juvenile offenders for two reasons. One is that only male juvenile offenders are adjudicated in this centralized juvenile reformatory, whereas the few female juvenile offenders are adjudicated in women’s prisons with other female adult offenders. The other reason is that focusing solely on male juvenile offenders is more suitable for the present study on chronic offending. Previous Western research that compared juvenile offending trajectories for male and female adolescents generally demonstrated that chronic offending is much more prevalent among male juvenile offenders [41,42]. In China, a study by Guo et al. (2017) reported gender differences in delinquency in Hubei province and found that only a small portion of all juvenile offenders are female. Guo et al. (2017) further provided detailed statistics on gender differences including the following particulars: (1) a district prosecutor’s office of Wuhan City handled 497 juvenile cases, of which only two (0.4%) involved female offenders; (2) among 100 juvenile offenders incarcerated in a juvenile reformatory in Hubei province, female offenders accounted for only 7% of all the juvenile offenders [43]. Given the small number of female juvenile offenders, it poses a challenge to collect data on chronic offending, a type of frequent offense over time, among female juvenile offenders in China. Thus, it is more suitable for the present study to focus solely on male juvenile offenders.

### 3.2. Data Collection Procedure

Considering the low level of literacy of respondents, this study adopted structured face-to-face interviews, which were consistent with the common practice in juvenile offender research [44]. Trained researchers explained the pre-developed questions to the juvenile offenders who expressed difficulty in understanding them. To prevent potential interference, all of the interviews were conducted in a private room within the juvenile reformatory. During the entire interview, only the respondents and researchers were permitted to be present. The duration of each interview averaged 45 min, and it took two weeks for the researchers to complete the data collection.

All of the ethics-related issues were assessed by the largest university in this autonomous region and were permitted by the authority in the juvenile reformatory. Before data collection, respondents in this research were informed that their participation was completely voluntary and anonymous and that non-participation or withdrawal due to discomfort during the interview would not lead to any negative consequences in their life in the reformatory. Data were collected by the researchers, who had received intensive six-hour training on interview skills, the content of the instrument, and security procedures to be followed during the interview.

At the time of the interviews, the juvenile reformatory incarcerated more than 1300 male juvenile offenders. Due to the concerns with memory decay and an insufficient sample size for analysis, the research participants were all the juvenile offenders who were admitted within 12 months before the interview. According to the juvenile reformatory’s official record, more than 800 juvenile offenders were eligible for the interview. Among them, more than a hundred were admitted and released within 12 months before the interview and, therefore, were not available for the interview. Thirteen of the youth did not participate due to illness or being in the segregation unit (*n* = 5), and eight declined to participate. The final sample size for the present study was 695. Because the missing values for all of the variables involved in the analysis were rather small, the listwise deletion method was utilized when conducting the statistical analysis.

### 3.3. Measurement

#### 3.3.1. Outcome Variable

The outcome variable is chronic offending. To distinguish chronic offending from other offending, this study identified three mutually exclusive groups: one-time offenders, recidivists, and chronic offenders. In line with the operationalization of Wolfgang’s study [45], one-time offenders were categorized as juvenile offenders with a history of only one offense, recidivists were categorized as juvenile offenders with a history of two to four offenses, and chronic offenders were categorized as juvenile offenders with a history of five or more offenses. The criminal history domain was constructed by measuring each juvenile offender’s total number of self-reported offenses in the current sample. The self-reported offenses include homicide, aggravated assault, molestation, rape, robbery, theft, fraud, arson, bombing, placing dangerous substances, dealing drugs, and other offenses.

#### 3.3.2. Predictor Variables

The predictor variables are the overall maltreatment victimization and five commonly identified child maltreatment types: physical abuse (PA), emotional abuse (EA), sexual abuse (SA), physical neglect (PN), and emotional neglect (EN). The short form of the Childhood Trauma Questionnaire (CTQ-SF) developed by Bernstein and his colleagues was utilized to measure child maltreatment [46]. The CTQ-SF includes 25 clinical items, which are classified as five subscales to measure the five maltreatment types. Each specific subscale consists of five items [46]. All items were prefaced by asking respondents: “Within the family context, have you ever experienced any of the following?” Example item statements include “I was punished with a belt, a board, a cord, or some other hard object” (PA); “People in my family called me things like ‘stupid’, ‘lazy’, or ‘ugly’” (EB); “Someone tried to make me do sexual things or watch sexual things” (SA); “My parents were too drunk or high to take care of the family” (PN); and “My family was a source of strength and support” (EN). The five items of emotional neglect were reverse coded to ensure consistency with the other four subscales. Each juvenile offender responded on a five-point Likert scale (1 = never true through 5 = very often true). The reliability Cronbach’s alpha values were 0.67, 0.69, 0.72, and 0.86 for the subscales of physical abuse, sexual abuse, physical neglect, and emotional neglect, respectively. The Cronbach’s alpha value of 0.57 for emotional abuse is relatively low, but this subscale is still retained because it has been considered an acceptable value in previous studies [15,47]. An overall maltreatment victimization score was created by summing up the total scores of 25 clinical items for each juvenile offender. Meanwhile, a specific score for each specific maltreatment type was created by calculating the mean score of the five items respectively in each subscale. The two scores represent the level of the overall maltreatment and each specific maltreatment type experienced by each juvenile offender. A higher score indicates that the offender experienced more serious maltreatment.

#### 3.3.3. Control Variables

To rule out potentially spurious effects of child maltreatment on chronic offending, related demographics and other well-established risk factors for chronic offending were included as control variables. In terms of related demographics, age and ethnicity were used as baseline demographic controls. Age was measured by the natural age that the respondent reported at the time of the interview, which was a continuous variable in the analysis. Ethnicity was coded as a dichotomous variable, with 1 indicating ethnic minority and 0 indicating Han ethnicity.

Individual-related risk factors included impulsivity and substance use. Impulsivity was coded as an interval variable by measuring the mean score of the four items: “I often act on the spur of the moment without stopping to think”, “I don’t devote much thought and effort to preparing for the future”, “I often do whatever brings me pleasure here and now, even at the cost of some distant goal”, and “I’m more concerned with what happens to me in the short run than in the long run” [48]. Each juvenile offender responded on a four-point Likert scale (1 = never true through 4 = always true; Cronbach’s alpha =  0.71), with higher scores representing greater impulsivity. Similarly, substance use was also coded as an interval variable by measuring the mean score of the following items: drinking alcohol, using marijuana, ecstasy, and other illegal drugs. Each juvenile offender was asked to report how many times they used the substance on a four-point Likert scale (1 =  never, 2 = one to two times, 3 = three to five times, 4 = six times or more), with higher scores representing more dependency.

Family-related risk factors included socioeconomic status and family structure. Socioeconomic status was measured by self-assessed family economic condition, ranging from 1 = affluent to 4 = poor. Family structure was measured by broken families and left-behind child families. Each juvenile offender was asked whether he has a broken family (divorce, separation, or death of parents) or whether he was left behind in his rural home by one parent or both parents due to the migration of the parent(s) to a city for work [49]. Responses were either 1, which indicates yes, or 0, which indicates no. Although being left behind by one’s family is not a well-established risk factor for chronic offending, it is considered a risk factor unique to the Chinese setting. Adolescents from such families were found to be more likely to engage in offending than their counterparts in previous Chinese research [50].

Peer-related risk factors included peer deviance and gang involvement. Peer deviance was assessed by using the peer delinquency scale [51], which comprises 25 nonoverlapping items to assess peer delinquent behaviors, including stealing, burglary, robbery, assault, and so on. Each juvenile offender was asked how many of his friends committed delinquent acts on a 5-point Likert-type scale (1 = none of them; 2 = a few of them; 3 = half of them; 4 = most of them; 5 = all of them; Cronbach’s alpha =  0.93). A higher mean score of the 25 items indicated a higher level of affiliation with delinquent peers. The gang involvement measure came from Zhang et al. [52] by summing up the total scores of the following three items for each juvenile offender: “doing illegal things is acceptable in your group”, “members of your group do illegal things together”, and “your group is a gang” (1 = yes, 0 = no), with higher scores representing higher levels of gang involvement.

Lastly, a school-related risk factor—namely, dropping out of school—was included as a control variable. Each juvenile offender was asked whether he had dropped out of school before committing the current offense. Responses were either 1 for yes or 0 for no.

### 3.4. Analytic Strategy

The analysis in this study can be broken down into two main parts. Descriptive analyses were performed to analyze the profile of the sample and explore the prevalence of child maltreatment victimization and chronic offending among juvenile offenders. Then, given the multinomial nature of the outcome variable (one-time offenders vs. recidivists vs. chronic offenders), multinomial logistic regression models were employed to examine the effect of child maltreatment on chronic offending.

## 4. Results

### 4.1. Descriptive Findings

#### 4.1.1. Descriptive Statistics on All of the Variables Included in the Analyses

Table 1 presents the descriptive statistics on all of the variables included in the analyses. Chronic offenders comprised 16.7% (*n* = 116) of the juvenile offender sample, while one-time offenders accounted for 53.4% (*n* = 371), and recidivists accounted for 29.9% (*n* = 208). Further analyses (not shown in Table 1) of the total number of offenses committed by the three groups of offenders show that chronic offenders reported 2333 counts of crimes in total, accounting for 71.9% of all of the crimes reported by the juvenile offender sample. The findings together revealed that a relatively small percentage (16.7%) of juvenile chronic offenders was responsible for the vast majority (71.9%) of the crimes committed by the whole sample.

The mean score of the overall maltreatment victimization was 41.27 (out of 125), which indicates that—in general—the juvenile offenders’ level of maltreatment victimization was not very high. Likewise, the mean scores of five specific maltreatment types ranged from as low as 1.14 (out of 5) for sexual abuse to as high as 2.53 (out of 5) for emotional neglect, indicating that the juvenile offenders in the sample perceived that they have experienced lower than moderate or moderate levels of child maltreatment. The highest level of maltreatment experienced was emotional neglect.

The demographic statistics showed that the mean age of juvenile offenders in the sample was 16.84 years old, ranging from 14 to 19 years. Ethnic minority juvenile offenders accounted for more than a third (*n* = 269, 38.7%) of the respondents, roughly the same proportion of the ethnic minority population in the region [40]. The descriptive statistics on the control variables show that the mean score of individual impulsivity was 2.11 out of 4, which indicates that these juvenile offenders had a medium level of trait impulsivity on average. Likewise, the mean score of substance use was 1.64 out of 4, indicating that the respondents did not portray themselves as highly dependent on drugs. On average, the juvenile offenders came from an average-income or low-income family (2.48 out of 4). More than a quarter (*n* = 195, 28.1%) came from broken families. Over half (*n* = 377, 54.2%) were left behind by one or both parents, which is a high proportion. The mean rating scores of peer deviance and gang involvement were 1.68 (out of 5) and 1.34 (out of 3), respectively, indicating that these juvenile offenders had a small number of friends who committed delinquent acts and that their own level of involvement in illegal gang activities was not high.

#### 4.1.2. Prevalence of Child Maltreatment Victimization among First-Time Offenders, Recidivists, and Chronic Offenders

Consistent with previous research [53], the following CTQ-SF cut-off scores were used to estimate the prevalence of child maltreatment victimization among juvenile offenders: physical abuse ≥ 8, emotional abuse ≥ 9, sexual abuse ≥ 6, physical neglect ≥ 8, emotional neglect ≥ 10. The cut-off scores were used to “capture cases with even the lowest severity of childhood trauma” [53] (p. 22). All of the prevalence estimations are displayed in Table 2.

Although the mean scores of both the overall maltreatment and specific maltreatment types were not high according to the aforementioned descriptive statistics, the result from using the cut-off scores shows that an overwhelming majority (93.2%) of the respondents had experienced maltreatment victimization. This high rate indicates that child maltreatment is rather common among Chinese juvenile offenders. In terms of exposure to five specific maltreatment types, there was wide variation in the juvenile offender sample, ranging from 17.0% for physical abuse to 85.0% for emotional neglect. The prevalence rates of the three remaining maltreatment subtypes—emotional abuse, sexual abuse, and physical neglect—were 25.3%, 24.3%, and 28.8%, respectively. Although physical abuse, sexual abuse, and emotional abuse were less prevalent than emotional neglect among the entire sample, they still occurred at relatively high rates. Moreover, both the overall prevalence of child maltreatment victimization and the prevalence rate of specific maltreatment types—except for physical abuse and physical neglect—seemed to be slightly higher among chronic offenders than among one-time offenders and recidivists (94% vs. 93.8% vs. 91.8% for overall prevalence).

### 4.2. Findings of Logistic Regression Analyses

Before conducting multinomial regression analysis, either ANOVA analyses or chi-square tests were performed to test group differences (one-time offenders, recidivists, and chronic offenders) in terms of independent variables and control variables. The result of the one-way ANOVA analysis revealed that there were no significant differences in overall maltreatment victimization among one-time offenders, recidivists, and chronic offenders (F = 0.01, *p* > 0.05). This finding indicates that overall maltreatment victimization was not related to chronic offending as compared to one-time offenders and recidivists. Further ANOVA analyses of the relationship between specific maltreatment types and offending types found significant group differences for physical abuse (F = 6.81, *p* < 0.01) and emotional abuse (F = 3.54, *p* < 0.05), indicating a need to conduct further regression analyses to test the relationships (see Table 3). Table 3 also presents group differences in the control variables among one-time juvenile offenders, recidivists, and chronic offenders. The results revealed significant group differences in gang involvement (χ^2^ = 4.01, *p* < 0.05) and school dropout (χ^2^ = 7.11, *p* < 0.05). The proportion of school dropout in chronic offenders was higher than that in one-time offenders and recidivists (87.93% vs.77.03% vs. 75.96%), which indicates that chronic offenders are significantly more likely to drop out of school.

Instead of performing only one multinomial logistic regression using chronic offenders as the reference group, two multinomial logistic regression models were created to (1) further explore the differential effects of five major maltreatment types on the three types of offenders and (2) facilitate explanation of the results. Table 4 shows the results of the two multinomial logistic regression models using one-time offenders as the reference group and recidivists as the reference group, respectively.

The results revealed no statistically significant difference in both child maltreatment subtypes and control variables when comparing one-time offenders with recidivists. When comparing chronic offenders with one-time offenders and recidivists, the results show that among the five major maltreatment types, only child physical abuse showed a statistically significant effect on chronic offending. Specifically, juvenile offenders who experienced a higher level of physical abuse were more likely to become chronic offenders than one-time offenders (OR = 3.90, *p* < 0.01) and recidivists (OR = 2.33, *p* < 0.05).

Among the control variables, both gang involvement and school dropout showed a significant effect on chronic offending. Specifically, juvenile offenders who were more involved in gang activities were more likely to become chronic offenders than one-time offenders (OR = 1.43, *p* < 0.01) and recidivists (OR = 1.52, *p* < 0.01). Meanwhile, compared with recidivists, juvenile offenders who dropped out of school before the current offense were more likely to become chronic offenders (OR = 2.32, *p* < 0.05).

Additional analyses of the chronic offender subsample show that among the 116 chronic offenders, a vast majority (*n* = 101, 87.1%) had committed at least one violent offense, including aggravated assault, sexual assault, and robbery. The result of the t test examining group differences in child maltreatment subtypes between non-violent chronic offenders and violent chronic offenders yielded a significant difference in only physical abuse between the two groups of chronic offenders (t = 2.13, *p* < 0.05). The mean score of physical abuse victimization among violent chronic offenders (M = 1.35) was significantly higher than that of non-violent chronic offenders, suggesting that child physical abuse may have a stronger negative impact on violent chronic offending than on non-violent chronic offending. Due to the small size of the subsample, however, no further regression analyses were conducted to investigate the effect of child maltreatment on violent chronic offending controlling for other related risk factors and demographics.

## 5. Discussion

Wolfgang et al., conducted a well-known study to explore patterns of chronic offending, known as “chronic 6%”, meaning a small percentage of juvenile chronic offenders who committed a large portion of overall crimes [31]. The chronic 6% were found among a birth cohort of school-aged children under age 18. The percentage reached 18% among the delinquent subset [32]. Although the overall delinquency rate has been found to be rather low in China, the present study found a similar pattern regarding chronic offending among juvenile offenders; that is, 16.7% of juvenile chronic offenders committed a vast majority of the overall reported crimes. This finding highlights the importance of studying juvenile chronic offending, a type of persistent offending that has been largely overlooked in previous delinquency research in China.

The finding that both the overall maltreatment victimization and the specific maltreatment victimization were prevalent among the juvenile offender sample is generally consistent with the results of Western studies that demonstrated a high incidence of child maltreatment victimization among juveniles involved in the justice system [4,5]. However, the prevalence rate of emotional neglect (85%) in the current sample is substantially higher than that of its Western counterparts (ranging from 34.8% to 39%) [6,8,54]. It may be attributed to several reasons. First, Chinese society emphasizes parental authority, which likely results in harsh discipline and a cold parent–child relationship in many Chinese families. Second, the high prevalence of emotional neglect may be explained by the large percentage (54.2%) of juvenile offenders who were left behind by one or both parents. As a result of urbanization processes, China is experiencing its largest internal migration in recent years. According to the 2021 National Bureau of Statistics of China, 17.9% or 252 million Chinese were children under the age of 15 in 2020 [40]. A recent international report further estimated that as many as 103 million Chinese children were affected by the massive internal migration. Of these children, more than 69 million were left-behind children [55]. The absence of parental supervision and care may reduce emotional bonding between parent(s) and children. Third, the high school dropout rate may also help explain the high prevalence of emotional neglect. In the current sample, the school dropout rate of juvenile offenders (78.8%) is rather high. Once juveniles drop out of school, they typically enter society early, and the time that they spend with their parents may be significantly reduced. Thus, parent–child emotional communication may be reduced.

Among the five maltreatment types, only physical abuse has been proven to exert a statistically significant impact on chronic offending among juvenile offenders. Moreover, its effect is stronger than the significant effects of other risk factors, including gang involvement and school dropout. The t test result comparing the mean scores of physical abuse victimization between violent chronic offenders and non-violent chronic offenders suggests that physical abuse victimization may also make a difference between the two types of chronic offending. The finding of the present study is partly consistent with that of Fox et al. [37] and Jung et al. [35], but it differs from that of Corrado and Peters [36]. While Corrado and Peters found no significant impact of physical abuse and sexual abuse on either general chronic offending or chronic violent offending [35]. Among juvenile offenders, Fox et al. discovered that not only physical abuse, but also emotional abuse and physical neglect, exerted a significant effect on chronic violent offending among juveniles [37]. The most recent study by Jung et al. revealed a significant relationship between physical–emotional abuse and sexual abuse and general chronic offending among juvenile offenders [35]. Along with the present study, it may be concluded that physical abuse likely exerts a significant effect on general chronic offending as well as violent chronic offending among juvenile offenders. However, this conclusion is tentative, and additional evidence is needed to confirm the finding.

The major finding on the relationship between physical child abuse victimization and chronic offending, especially violent chronic offending, provides empirical support for Agnew’s general strain argument that physical abuse as one of the major stressful life events may lead to juvenile delinquency [20,21], including chronic offending. Meanwhile, the finding also lends support to social learning theory and the “cycle of violence” argument on the relationship between child physical abuse and the perpetuation of violence [23,24]—that is, juvenile chronic offending, especially violent chronic offending, is a behavior that is learned and reinforced from the abusive behavior, especially physical abuse, of the parents or other family members.

The findings together have important social policy implications. First, the high prevalence of child maltreatment among juvenile offenders indicates a need for prevention programs provided to maltreated children in order to tackle delinquency in general. Second, the finding that physical abuse is a major risk factor for chronic offending, especially violent chronic offending, highlights the importance of intervention programs providing treatment to the physically abused children after they have manifested their initial criminal acts.

Meanwhile, the findings call into question the traditional parenting style of Chinese families. Owing to the importance of filial piety as a moral principle, Chinese society tends to emphasize parental authority. Obeying and respecting their parents are appropriate standards for children in Chinese families [56]. In this “controlling” or “authoritarian” context [57], parental authority and children’s obedience are typically maintained through harsh discipline, which is reflected in the well-known proverb “spare the rod, spoil the child”. Such parental practices may result in the blurring of boundaries between discipline and physical abuse. Despite a shift toward a combination of authoritative, egalitarian, and care-oriented parenting styles in recent years [58], this study indicates that certain harsh disciplinary practices that are traditionally acceptable in China continue to exist and should be reconsidered. Comparatively, the relationship between parenting styles and children’s behavior has been extensively researched in the West. In the 1960s, Baumrind (1966) [59] developed the well-known theory on parenting styles which was later refined by Maccoby and Martin (1983) [60] and many other scholars in their empirical research. The theory identifies four major types of parenting styles that exist among many parents—authoritative, authoritarian, permissive, and neglectful parenting styles—based on evaluation on parental responsiveness and demandingness. Among them, authoritarian parenting style that prioritize a high level of control over children has been found to be associated with children’s externalizing problems including delinquency [61,62]. A recent study that compared parenting practices and their influence on juvenile delinquency between China and the United States revealed both similarities and significant differences in parenting practices between the two countries and highlighted “the need to consider cultural differences when assessing the impact of parenting practices on delinquency” [63] (p. 1). Future research should take this into consideration and explore in more depth the relationship between specific parenting styles and juvenile chronic offending in different cultural context.

This study has several limitations that should be addressed in future research. First, despite the relationship found between child physical abuse victimization and juvenile chronic offending, the cross-sectional data do not confirm the causal relationship between physical abuse and chronic offending. Second, the sample is drawn from a provincial-level juvenile reformatory in southwestern China, which limits the generalizability of the findings. Third, although previous studies have shown that using self-reported methods to measure chronic offending is feasible [64,65], juvenile offenders may encounter difficulty in understanding what constitutes criminal behavior due to a low level of literacy. As a result, they may misreport their criminal behavior. Meanwhile, they may overreport or underreport their crimes due to either deliberate deception or self-preservation [66]. In addition, the structured interview questions did not cover details regarding the family member(s) who abused the juvenile respondents. This poses a constraint to further explore the relationship between the investigated juvenile offenders and their parents as well as their other family members. Future research should take all of these limitations into consideration by making use of large-scale longitudinal data and data from other sources, such as official police data, to ensure the validity of the results.

## 6. Conclusions

Despite the limitations, the present study provides a rare opportunity to gain a comprehensive understanding of child maltreatment victimization and chronic offending among juvenile offenders in China. In particular, it explored both prevalence of child maltreatment victimization and applicability of the chronic offending concept among juvenile offenders in the Chinese context. Furthermore, it offers a valuable first step toward tapping simultaneously into the holistic effect of the overall maltreatment victimization and the differential effects of major maltreatment types on chronic offending. The evidence presented in this study clearly demonstrates a relationship between child physical abuse victimization and chronic offending, including violent chronic offending, among Chinese juvenile offenders. More research is needed to corroborate this relationship in the future.

## Figures and Tables

**Table 1:** Descriptive analysis of all variables included in the analyses (N = 695).

	Variables	Range	% ^a^ or M ^b^	*n* ^a^ or SD ^b^
Outcome variables	One-time offenders (No. offenses = 1)	0, 1 ^c^	53.4%	371
	Recidivists (2 ≤ No. offenses < 5)	0, 1 ^c^	29.9%	208
	Chronic offenders (No. offenses ≥ 5)	0, 1 ^c^	16.7%	116
Predictor variables	Overall maltreatment victimization	25–125	41.27	6.98
	Physical abuse	1–5	1.21	0.39
	Emotional abuse	1–5	1.47	0.51
	Sexual abuse	1–5	1.14	0.34
	Physical neglect	1–5	1.41	0.65
	Emotional neglect	1–5	2.53	1.21
Demographics	Age	14–19	16.84	0.97
	Ethnicity	0, 1		
	Ethnic minority	1	38.7%	269
	Han ethnicity	0	61.3%	426
Individual-related risk factors	Impulsivity	1–4	2.11	0.78
	Substance Use	1–4	1.64	0.58
Family-related risk factors	Family economic condition	1–4	2.48	0.73
	Broken family	0, 1		
	Yes	1	28.1%	195
	No	0	71.9%	500
	Left-behind child family	0, 1		
	Yes	1	54.2%	377
	No	0	45.8%	318
Peer-related risk factors	Peer deviance	1–5	1.68	0.57
	Gang involvement	0–3	1.34	1.10
School-related risk factor	Dropped out of school	0, 1		
	Yes	1	78.8%	548
	No	0	21.2%	147

Note: N, total sample size; M, mean; *n*, frequency; SD, standard deviation; ^a^ calculated for binary or categorical variables; ^b^ calculated for interval or ordinal variables; ^c^ value depends on if the relevant category is estimated as a reference group in the multinomial logistic regression.

**Table 2:** Prevalence of child maltreatment victimization for the overall sample and various offender subgroups (based on cut-off scores).

Maltreatment Victimization		Overall Sample (N = 695)	One-Time Offender (*n* = 371)	Recidivist (*n* = 208)	Chronic Offender (*n* = 116)
		*n* (%)	*n* (%)	*n* (%)	*n* (%)
Overall maltreatment victimization		648 (93.2%)	348 (93.8%)	191 (91.8%)	109 (94.0%)
Specific maltreatment types	Physical abuse	118 (17.0%)	59 (15.9%)	39 (18.8%)	20 (17.2%)
	Emotional abuse	176 (25.3%)	97 (26.1%)	48 (23.1%)	31 (26.7%)
	Sexual abuse	169 (24.3%)	94 (25.3%)	45 (21.6%)	30 (25.9%)
	Physical neglect	200 (28.8%)	106 (28.6%)	62 (29.8%)	32 (27.6%)
	Emotional neglect	591 (85.0%)	316 (85.2%)	174 (83.7%)	101 (87.1%)

**Table 3:** Group differences in the independent variables and control variables.

	Variables	One-Time Offenders		Recidivists		Chronic Offenders		
		Mean or *n*	SD or %	Mean or *n*	SD or %	Mean or *n*	SD or %	F or χ^2^
Specific maltreatment types	Physical abuse	1.18	0.39	1.21	0.35	1.33	0.44	6.81 **
	Emotional abuse	1.45	0.53	1.55	0.62	1.60	0.62	3.54 *
	Sexual abuse	1.12	0.31	1.15	0.39	1.17	0.34	1.03
	Physical neglect	1.40	0.62	1.36	0.64	1.51	0.72	1.86
	Emotional neglect	2.46	1.20	2.55	1.24	2.73	1.20	2.26
Individual-related risk factors	Impulsivity	2.13	0.79	2.12	0.77	2.00	0.76	1.43
	Substance use	1.63	0.58	1.66	0.63	1.61	0.49	0.25
Family-related risk factors	Family economic condition	2.51	0.78	2.43	0.67	2.48	0.69	0.88
	Broken family							1.73
	Yes	97	26.15%	65	31.25%	33	28.45%	
	No	274	73.85%	143	68.75%	83	71.55%	
	Left-behind child family							0.12
	Yes	199	53.64%	114	54.81%	64	55.17%	
	No	172	46.36%	94	45.19%	52	44.83%	
Peer-related risks factor	Peer deviance	1.66	0.57	1.67	0.56	1.72	0.60	0.35
	Gang involvement	1.29	1.07	1.25	1.14	1.65	1.08	4.01 *
School-related risk factor	Dropped out of school							7.11 *
	Yes	288	77.63%	158	75.96%	102	87.93%	
	No	83	22.37%	50	24.04%	14	12.06%	
Demographics	Age	16.81	0.99	16.83	0.96	16.96	0.95	1.04
	Ethnicity							4.60
	Ethnic minority	131	35.31%	85	40.87%	53	45.69%	
	Han ethnicity	240	64.69%	123	59.13%	63	54.31%	

Note: * *p* < 0.05; ** *p* < 0.01.

**Table 4:** Multinomial logistic regression results on the effect of maltreatment subtypes across offender types (N = 695).

Variables		Model 1				Model 2			
		Recidivist		Chronic Offender		One-Time Offender		Chronic Offender	
		vs. One-Time Offender (Reference)				vs. Recidivist (Reference)			
		b (S.E.)	OR (95% CI)	b (S.E.)	OR (95% CI)	b (S.E.)	OR (95% CI)	b (S.E.)	OR (95% CI)
Specific maltreatment types	Physical abuse	0.52 (0.32)	1.68 (0.90–3.12)	1.36 (0.34)	3.90 ** (2.00–7.62)	−0.52 (0.32)	0.60 (0.32–1.11)	0.85 (0.36)	2.33 * (1.16–4.68)
	Emotional abuse	0.01 (0.21)	1.01 (0.67–1.52)	−0.05 (0.28)	0.96 (0.55–1.66)	−0.01 (0.21)	1.00 (0.66–1.50)	−0.05 (0.30)	0.95 (0.53–1.71)
	Sexual abuse	0.02 (0.31)	1.02 (0.56–1.85)	−0.10 (0.38)	0.91 (0.43–1.92)	−0.02 (0.31)	0.98 (0.54–1.78)	−0.12 (0.40)	0.89 (0.41–1.96)
	Physical neglect	−0.11 (0.18)	0.89 (0.63–1.26)	−0.14 (0.21)	0.87 (0.57–1.32)	0.11 (0.18)	1.12 (0.80–1.58)	−0.03 (0.23)	0.97 (0.61–1.54)
	Emotional neglect	0.05 (0.09)	1.06 (0.89–1.25)	0.08 (0.11)	1.08 (0.87–1.35)	−0.05 (0.09)	0.95 (0.80–1.12)	0.03 (0.12)	1.03 (0.82–1.30)
Individual-related risk factors	Impulsivity	0.06 (0.14)	1.07 (0.81–1.40)	−0.24 (0.19)	0.79 (0.54–1.15)	−0.06 (0.14)	0.94 (0.72–1.23)	−0.30 (0.21)	0.74 (0.50–1.11)
	Substance use	0.26 (0.20)	1.30 (0.88–1.90)	−0.24 (0.27)	0.79 (0.47–1.33)	−0.26 (0.20)	0.77 (0.53–1.13)	−0.50 (0.28)	0.61 (0.35–1.06)
Family-related risk factors	Family economic condition	−0.16 (0.15)	0.85 (0.64–1.14)	0.16 (0.19)	1.17 (0.81–1.69)	0.16 (0.15)	1.17 (0.87–1.57)	0.32 (0.21)	1.37 (0.92–2.06)
	Broken family	0.30 (0.24)	1.34 (0.85–2.13)	−0.10 (0.31)	0.90 (0.49–1.66)	−0.30 (0.24)	0.75 (0.47–1.18)	−0.40 (0.33)	0.67 (0.35–1.29)
	Left-behind child family	−0.05 (0.21)	0.96 (0.63–1.44)	0.22 (0.28)	1.25 (0.73–2.15)	0.05 (0.21)	1.05 (0.69–1.58)	0.27 (0.30)	1.31 (0.73–2.34)
Peer-related risk factors	Peer deviance	−0.16 (0.22)	0.86 (0.56–1.31)	0.11 (0.28)	1.12 (0.65–1.92)	0.16 (0.22)	1.17 (0.76–1.79)	0.27 (0.30)	1.31 (0.73–2.36)
	Gang involvement	−0.06 (0.11)	0.94 (0.77–1.16)	0.36 (0.14)	1.43 ** (1.10–1.87)	0.06 (0.11)	1.06 (0.86–1.31)	0.42 (0.15)	1.52 ** (1.14–2.02)
School-related risk factor	Dropped out of school	−0.18 (0.24)	0.83 (0.52–1.33)	0.66 (0.38)	1.94 (0.92–4.08)	0.18 (0.24)	1.20 (0.75–1.91)	0.84 (0.40)	2.32 * (1.07–5.06)
Demographics	Age	−0.01 (0.11)	0.99 (0.80–1.22)	0.13 (0.14)	1.14 (0.86–1.51)	0.01 (0.11)	1.01 (0.82–1.25)	0.14 (0.16)	1.15 (0.85–1.56)
	Ethnicity	0.24 (0.21)	1.27 (0.83–1.93)	0.23 (0.27)	1.25 (0.73–2.14)	−0.24 (0.21)	0.79 (0.52–1.20)	−0.01 (0.30)	0.99 (0.55–1.77)
**Pseudo R^2^**	10.7%								

Note: * *p* < 0.05; ** *p* < 0.01; b, b coefficient; OR, odds ratio; S.E., standard error; CI, confidence interval.

## Data Availability

Data sharing is not applicable to this article.
